# Global burden and trends of chronic kidney disease due to type 2 diabetes mellitus caused by dietary risks: insights from the global burden of disease study 2021

**DOI:** 10.3389/fendo.2025.1623795

**Published:** 2025-10-06

**Authors:** Xiaoyue Wang, Yunfeng Yu, Gang Hu, Xinyu Yang, Yuman Yin, Junju Zou, Rong Yu

**Affiliations:** ^1^ School of Traditional Chinese Medicine, Hunan University of Chinese Medicine, Changsha, Hunan, China; ^2^ Department of Pediatrics, The First Hospital of Hunan University of Chinese Medicine, Changsha, Hunan, China; ^3^ Department of Endocrine, The First Hospital of Hunan University of Chinese Medicine, Changsha, Hunan, China

**Keywords:** dietary risks, chronic kidney disease, type 2 diabetes mellitus, global burden of disease, socio-demographic index, healthcare

## Abstract

**Objective:**

The burden of dietary risk-induced diseases, including chronic kidney disease due to type 2 diabetes mellitus caused by dietary risks (CKD-T2DM-DR), has been consistently underestimated. This study aims to investigate the burden of CKD-T2DM-DR from 1990 to 2021 globally and regionally.

**Methods:**

The global burden of disease (GBD) database served as the data source for analyzing the mortality, agestandardized mortality rate (ASMR), disability-adjusted life years (DALYs), age standardized DALY rate (ASDR), and estimated annual percentage change (EAPC) of CKD-T2DM-DR worldwide from 1990 to 2021. Subsequently, the ASMR, ASDR, and EAPC were further evaluated in different regions, age, gender, and socio-demographic index (SDI) groups. Finally, the burden of CKD-T2DM-DR induced by different dietary risks was reported and compared.

**Results:**

Globally, the mortality, ASMR, DALYs, and ASDR of CKD-T2DM-DR were on the rise from 1990 to 2021. The global mortality of CKD-T2DM-DR in 2021 was 79,988 (95% uncertainty interval [UI] 32,734–128,884), ASMR was 0.96 (95% UI 0.4–1.54) per 100,000 population, DALYs were 1,999,209 (95% UI 856,194–3,167,215), and ASDR was 23.21 (95% UI 9.95–36.61) per 100,000 population. Regionally, low SDI regions exhibited the highest ASDR (27.41 [95% UI 11.32–46.78] per 100,000 population) and ASMR (1.16 [95% UI 0.44–2.02] per 100,000 population), whereas high-middle SDI regions recorded the lowest ASDR (14.7 [95% UI 5.96–23.77] per 100,000 population) and ASMR (0.59 [95% UI 0.24–0.97] per 100,000 population). High SDI regions presented a rapid increase in ASDR and ASMR, with EAPCs of 1.02 (95% CI 0.86–1.19) and 1.4 (95% CI 1.23–1.58), respectively. The correlation analysis supported that ASDR and ASMR were negatively associated with SDI. Additionally, the global burden of CKD-T2DM-DR increased with age and was higher in men than in women. Among the seven associated dietary risks, the DALY and death percents of CKD-T2DM caused by diet low in fruit were the highest, at 4.57% and 4.31%, respectively.

**Conclusion:**

The global burden of CKD-T2DM-DR has been steadily increasing with significant regional variability. Low SDI regions are most severely affected by this challenge, while high SDI regions are experiencing a rapid increase in the burden. The diet low in fruit was identified as the primary dietary risk for CKD-T2DM. This highlights the urgent need for rapid growth in the targeted prevention and health care strategies to alleviate the global burden of CKD-T2DM-DR.

## 1 Introduction

Diabetic kidney disease (DKD) is a chronic kidney disease (CKD) induced by hyperglycemia and is among the most prevalent complications associated with diabetes (1). The prevalence of diabetes and its complications pose a significant threat to global health. Epidemiological surveys reveal that the population of adult patients with diabetes worldwide reached 588.7 million in 2024, with forecasts suggesting an increase to 852.5 million by 2050 (2). In the United States, approximately 34.2 million individuals are affected by diabetes, while in China, the figure stands at 149 million, constituting 10.5% and 10.9% of their respective populations (3, 4). According to the International Diabetes Federation, up to 40% of people with diabetes suffer from DKD (2). The prevalence of DKD in people with diabetes varies dramatically between countries, ranging from 27.1% in China to 83.6% in Tanzania (5). Although the global prevalence of DKD has remained relatively stable, the continuous growth of the population worldwide means that the absolute number of people with DKD is increasing (6). DKD represents the most serious complication of diabetes and the primary cause of end-stage kidney disease (ESKD) (7), which accounts for 47% of all ESKD cases in the United States and more than 60% in Malaysia (8). It is reported that the annual expenditure of the United Kingdom National Health Service on the DKD is $1.2 billion (9), and the total medical expenditure on the management of DKD in the United States is as high as $16.8 billion per year (9). DKD imposes a significant economic burden on families and society, and has emerged as a pressing global public health concern.

Previous views believed that diabetes, hyperlipidemia, and hyperuricemia are significant risk factors for DKD, which are closely linked to dietary factors (7, 10). Subsequent studies demonstrated that dietary structures, such as high red meat diets and low fibre diets, influence the incidence and prognosis of DKD. In the United States, a cross-sectional study showed that dietary fibre intake was negatively associated with the risk of DKD (odds ratio [OR] 0.89, 95% confidence interval [CI] 0.80–0.99) (11). Another case-control study in Iran reported that a diet high in red meat increased the risk of DKD by 181% (OR 2.81, 95% CI 1.09–7.21) (12). However, the global prevalence and mortality of DKD attributable to dietary risks remain largely unknown. Moreover, previous studies have not systematically examined the differences in DKD caused by dietary risks (DKD-DR) across socio-demographic index (SDI) categories or regional disparities. These knowledge gaps make it challenging for epidemiologists and policymakers to accurately evaluate the health burden attributable to dietary risks, particularly in developing and underdeveloped regions. Therefore, this study aims to systematically quantify the global, regional, and SDI-specific burden and determinants of DKD-DR, thereby informing evidence-based prevention and management strategies.

The global burden of disease (GBD) study gathers epidemiological data from 204 countries/territories worldwide, providing a valuable platform for evaluating the impact of diseases on health (13). GBD 2021 data allows standardized comparisons across 204 countries and territories, effectively overcoming the issue of insufficient epidemiological data in developing areas, thereby supporting unified cross-regional comparisons and analyses. Given that type 2 diabetes mellitus (T2DM) is the most common risk factor leading to DKD, we utilized GBD 2021 data in this study to assess the burden and trends of chronic kidney disease due to type 2 diabetes mellitus caused by dietary risks (CKD-T2DM-DR). Through this meticulous analysis, we aimed to provide medical institutions and policymakers with a reliable reference to address the challenges posed by CKD-T2DM-DR.

## 2 Methods

### 2.1 Study design

This study analyzed the mortality, age-standardized mortality rate (ASMR), disability-adjusted life years (DALYs), age-standardized DALY rate (ASDR), and estimated annual percentage changes (EAPCs) of CKD-T2DM-DR worldwide using the GBD database. Moreover, it reveals the global burden, trends, and influencing factors of CKD-T2DM-DR by collating the relevant data of CKD-T2DM-DR in different regions, SDI, gender, and age groups.

### 2.2 Data source

In this study, CKD-T2DM-DR refers to CKD attributable to T2DM, with its burden specifically assigned to dietary risks based on the GBD risk attribution framework. GBD estimates the burden of CKD-T2DM-DR using a counterfactual comparative risk assessment. For each dietary factor, the TMREL is independently defined without assuming changes in co-occurring exposures. To account for overlapping effects and mediated pathways among multiple risks, GBD applies a mediation matrix, which avoids overestimating the combined burden and ensures consistency in calculating PAFs. Therefore, the CKD-T2DM-DR estimates in this study reflect the independent impact of dietary factors, excluding confounding influences from other risks.

The data were obtained from the GBD 2021 database via the Global Health Data Exchange (vizhub.healthdata.org) (14). The CKD-T2DM dataset was extracted from “chronic kidney disease due to diabetes mellitus type 2” within “chronic kidney disease” under “diabetes and kidney diseases” and was coded as E11.2 according to ICD-10. This study focused on individuals aged 25+ and included gender-specific subgrouping (male/female). Seven risks associated with CKD-T2DM were analyzed: diet high in processed meat, diet high in red meat, diet high in sodium, diet high in sugar-sweetened beverages, diet low in fruits, diet low in vegetables, and diet low in whole grains. As the GBD data are publicly available and anonymized, no additional ethical approval was required.

### 2.3 Statistical analysis

R 4.4.1 was used for data analysis and visualization. We assessed the global burden and temporal trends of CVD-DR according to the mortality, DALYs, ASMRs, ASDRs, and EAPCs. The deaths were defined as the number of individuals who died of CVD-DR in a given year. DALY was calculated as the sum of years of life lost (YLL) due to premature mortality and years lived with disability (YLD) due to disease, which reflected the overall disease burden, as follows: DALY = YLL + YLD. We determined the age-standardized rate (ASR) as the weighted average of age-specific rates, where the weights were the proportions of each age group in a standard population, including the ASMR and ASDR. ASMR refers to the age-standardized rate per 100,000 deaths, and ASDR as the age-standardized DALY rate per 100,000 population. We calculated the ASR using the following formula: $\text{ASR}=\sum_{i=1}^{A}a_{i}w_{i}/\sum_{i=1}^{A}w_{i}\times 100,000$ , where *a_i_* represents the *i-*th age group and *w_i_* is the number (or proportion) of the population in the same age group in the GBD world standard population. All estimates are presented with 95% uncertainty intervals (UIs) derived from the GBD 2021 study framework. These UIs represent Bayesian credible intervals calculated from 1,000 posterior draws that incorporate uncertainties from incomplete data, model assumptions, and measurement errors. The 2.5th and 97.5th percentiles of the ordered draws are considered the lower and upper bounds, respectively, indicating a 95% probability that the true value lies within this range. The EAPC quantifies the average annual percentage change in ASMR or ASDR over a specified period. We assessed these temporal trends using a linear regression model, based on the equation Y = α + βX + ϵ, where Y is the natural logarithm of the ASR, X denotes the calendar year, and ϵ is the error term. We calculated EAPC using the formula EAPC = 100 × (exp (β) - 1), which was presented as numerical values and 95% confidence intervals (CIs). EAPC > 0 indicated an increasing trend, whereas EAPC< 0 indicated a decreasing trend.

Subsequently, we analyzed ASMR and ASDR for CKD-T2DM-DR across regions, SDI, gender, and age groups to further evaluate factors influencing disease burden. The regional subgroup analysis assessed the burden across 21 regions and 204 countries or territories. SDI, a composite indicator reflecting socioeconomic development, integrates per capita income, educational attainment, and fertility rate, with higher values representing greater socioeconomic advancement. Countries and territories were classified into five SDI categories, and the association between disease burden and SDI was evaluated using the Spearman correlation coefficient (*ρ*). A *ρ* value closer to 1 indicates a strong positive monotonic relationship, whereas a value closer to −1 reflects a strong negative monotonic relationship. Statistical significance was set at *p* < 0.05. Gender subgroup analyses evaluated disease burden separately for males and females, while age subgroup analyses divided the population into 15 groups in five-year intervals, starting from 25 years and above. Finally, the primary dietary risk was identified by analyzing the global proportions of DALY and death attributed to CKD-T2DM-DR.

## 3 Results

### 3.1 Mortality and ASMR

From 1990 to 2021, the global mortality attributed to CKD-T2DM-DR increased from 27,232 (95% UI 11,101–42,889) to 79,988 (95% UI 32,734–128,884), and the ASMR increased from 0.78 (95% UI 0.31–1.23) per 100,000 population to 0.96 (95% UI 0.4–1.54) per 100,000 population, with an EAPC of 0.74 (95% CI 0.58–0.91), as shown in **Table 1**.

**Table 1:** Death cases and ASMR of CKD-T2DM-DR and its temporal trends from 1990–2021.

Location	Death cases (95% UI)		ASMR (95% UI)		1990–2021 EAPCs (95% CI)
	1990	2021	1990	2021	
Global	27,232 (11,101–42,889)	79,988 (32,734–128,884)	0.78 (0.31–1.23)	0.96 (0.4–1.54)	0.74 (0.58–0.91)
SDI					
High SDI	7,834 (3,582–11,801)	24,874 (10,721–38,332)	0.7 (0.32–1.07)	1.06 (0.45–1.61)	1.4 (1.23–1.58)
High-middle SDI	4,893 (1,864–8,065)	11,428 (4,580–18,953)	0.57 (0.22–0.93)	0.59 (0.24–0.97)	0.06 (-0.17–0.29)
Middle SDI	7,922 (2,954–13,163)	25,470 (9,852–42,812)	0.94 (0.35–1.59)	1.03 (0.4–1.73)	0.43 (0.27–0.58)
Low-middle SDI	4,352 (1,724–7,193)	13,260 (5,431–22,589)	0.84 (0.32–1.4)	1.01 (0.41–1.72)	0.69 (0.54–0.84)
Low SDI	2,204 (890–3,660)	4,894 (1967–8,490)	1.15 (0.46–1.92)	1.16 (0.44–2.02)	0.01 (-0.1–0.13)
Central Europe, eastern Europe, and central Asia					
Central Asia	102 (52–159)	309 (150–479)	0.22 (0.11–0.35)	0.41 (0.2–0.64)	1.36 (0.93–1.78)
Central Europe	492 (235–773)	685 (308–1,123)	0.35 (0.16–0.54)	0.29 (0.13–0.47)	-0.58 (-1.05–-0.11)
Eastern Europe	476 (205–731)	903 (426–1,407)	0.17 (0.07–0.26)	0.25 (0.12–0.39)	0.58 (0.31–0.84)
High income region					
High-income Asia Pacific	2,409 (1,221–3,573)	5,557 (2,414–8,756)	1.34 (0.68–1.99)	0.86 (0.37–1.31)	-1.57 (-1.88–-1.27)
High-income North America	2,666 (1,111–4,157)	12,934 (4,944–19,978)	0.74 (0.31–1.15)	1.9 (0.71–2.91)	3.17 (2.97–3.37)
Western Europe	2,792 (1,263–4,413)	5,753 (2,549–9,584)	0.46 (0.2–0.75)	0.46 (0.2–0.75)	0.36 (0.1–0.63)
Australasia	40 (18–63)	144 (57–248)	0.18 (0.08–0.29)	0.24 (0.1–0.41)	1.62 (1.33–1.92)
Southern Latin America	632 (228–1,087)	1,001 (400–1,662)	1.44 (0.52–2.47)	1.11 (0.44–1.84)	-0.47 (-0.8–-0.14)
Latin America and Caribbean					
Andean Latin America	285 (96–505)	1,149 (419–2,077)	1.55 (0.51–2.77)	2.01 (0.73–3.63)	0.95 (0.8–1.09)
Caribbean	321 (129–534)	924 (401–1,603)	1.32 (0.52–2.2)	1.7 (0.74–2.95)	1.39 (1.12–1.65)
Central Latin America	838 (361–1,411)	4,391 (1,894–7,257)	1.17 (0.49–1.99)	1.79 (0.76–2.97)	2.04 (1.72–2.35)
Tropical Latin America	1,094 (456–1,762)	3,945 (1,671–6,321)	1.38 (0.57–2.25)	1.57 (0.66–2.52)	0.41 (0.09–0.74)
North Africa and Middle East					
North Africa and Middle East	1,227 (462–2,188)	3,009 (1,149–5,153)	0.86 (0.32–1.54)	0.73 (0.28–1.24)	-0.55 (-0.72–-0.38)
South Asia					
South Asia	3,886 (1,623–6,619)	13,037 (5,227–23,153)	0.79 (0.32–1.34)	0.96 (0.37–1.68)	0.64 (0.46–0.82)
Southeast Asia, east Asia, and Oceania					
East Asia	6,156 (1,839–10,785)	16,125 (5,177–28,752)	0.95 (0.3–1.67)	0.82 (0.26–1.43)	-0.55 (-0.75–-0.35)
Oceania	26 (7–50)	83 (26–151)	1.13 (0.32–2.1)	1.38 (0.43–2.5)	0.56 (0.43–0.7)
Southeast Asia	1,678 (522–3,130)	5,233 (1,554–10,117)	0.79 (0.25–1.48)	0.9 (0.27–1.74)	0.52 (0.48–0.56)
Sub-Saharan Africa					
Eastern Sub-Saharan Africa	1,117 (452–1,965)	2,634 (1,113–4,467)	1.81 (0.7–3.15)	1.99 (0.81–3.37)	0.19 (0.08–0.3)
Central Sub-Saharan Africa	285 (105–504)	591 (207–1,065)	1.59 (0.55–2.76)	1.37 (0.45–2.54)	-0.78 (-0.97–-0.59)
Southern Sub-Saharan Africa	125 (48–221)	341 (137–594)	0.52 (0.19–0.93)	0.67 (0.26–1.19)	1.29 (0.8–1.77)
Western Sub-Saharan Africa	584 (189–1,021)	1,241 (444–2,146)	0.8 (0.26–1.41)	0.77 (0.27–1.33)	-0.19 (-0.3–-0.09)

CKD-T2DM-DR, chronic kidney disease due to type 2 diabetes mellitus caused by dietary risks; ASMR, age-standardized mortality rate; EAPCs, estimated annual percentage changes. When EAPC > 0, ASMR or ASDR tends to increase; when EAPC< 0, ASMR or ASDR tends to decrease.

#### 3.1.1 Regional variations analyses

**Figure 1:**
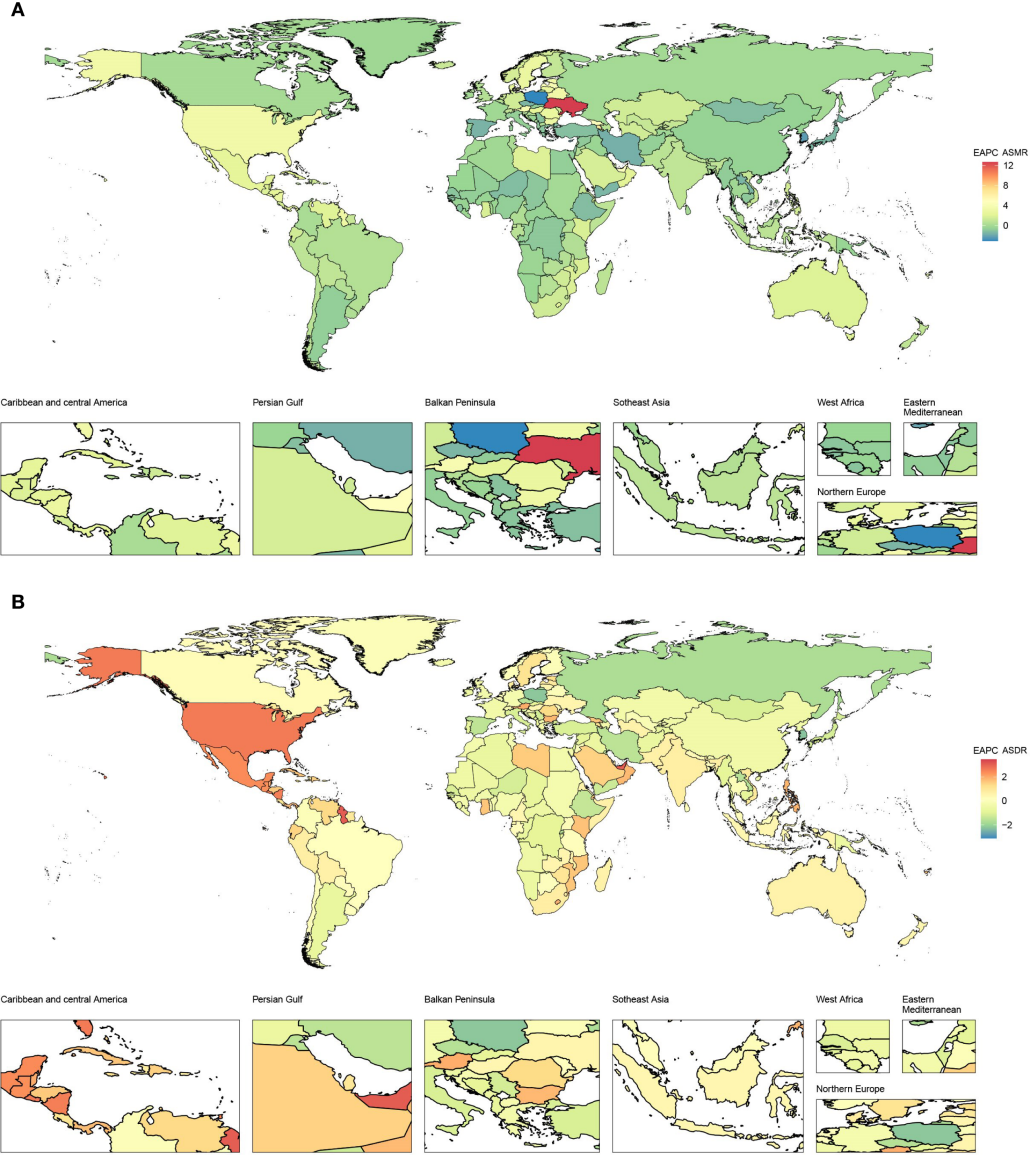
EAPCs of CKD-T2DM-DR in 204 countries or territories from 1990 to 2021. **(A)** EAPC of ASMR; **(B)** EAPC of ASDR. CKD-T2DM-DR, chronic kidney disease due to type 2 diabetes mellitus caused by dietary risks; EAPCs, estimated annual percentage changes; ASMR, age-standardized mortality rate; ASDR, age-standardized disability-adjusted life years rate. When EAPC > 0, ASMR or ASDR tends to increase; when EAPC< 0, ASMR or ASDR tends to decrease.

**Figure 2:**
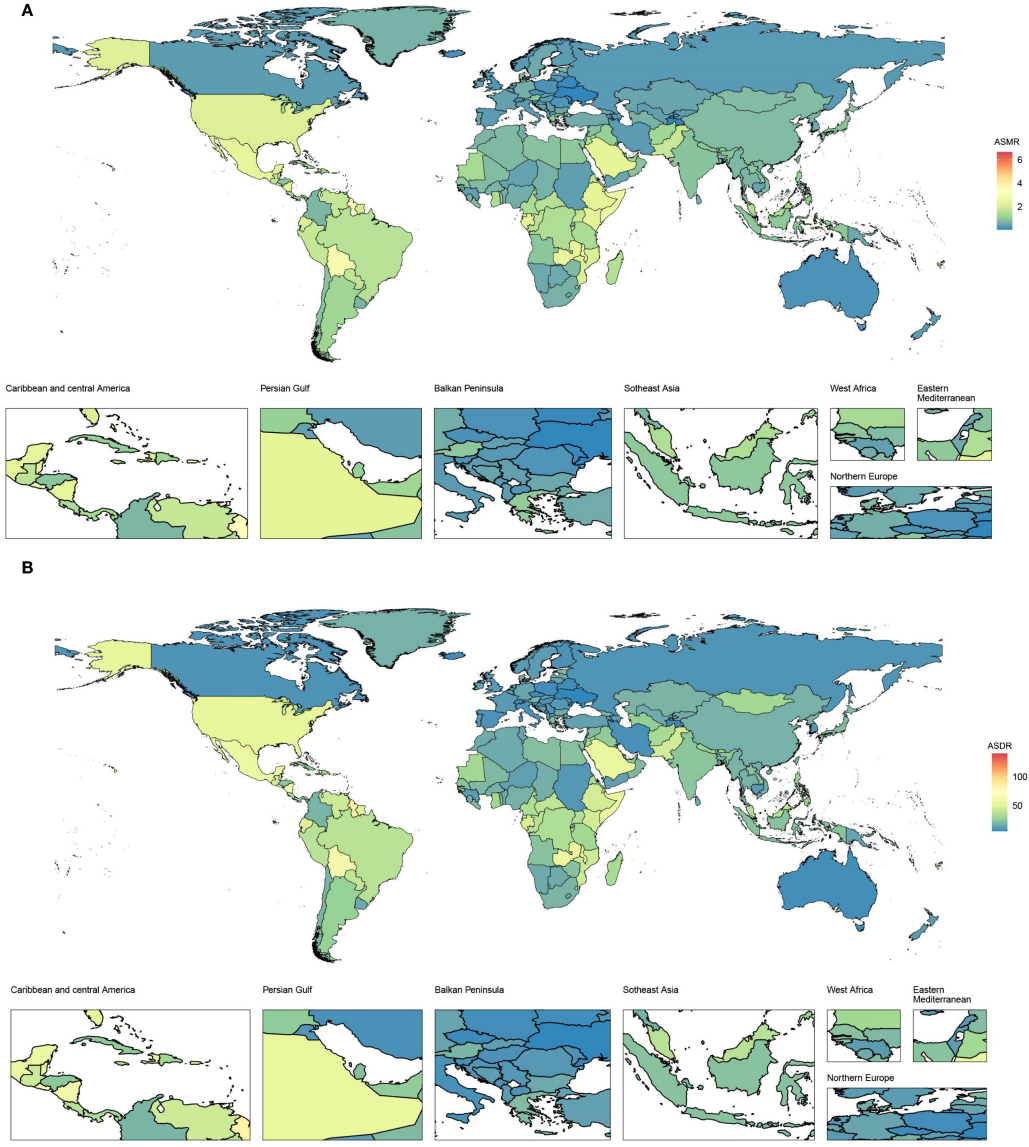
Age-standardized rates of CKD-T2DM-DR in 204 countries or territories in 2021. **(A)** ASMR; **(B)** ASDR. CKD-T2DM-DR, chronic kidney disease due to type 2 diabetes mellitus caused by dietary risks; ASMR, age-standardized mortality rate; ASDR, age-standardized disability adjusted life years rate.

From 1990 to 2021, ASMR in high-income North America increased the fastest, with an EAPC of 3.17 (95% CI 2.97–3.37), and ASMR in high-income Asia Pacific decreased the fastest, with an EAPC of -1.57 (95% CI -1.88 to -1.27), as depicted in **Figure 1A** and **Table 1**. In 2021, ASMR varied markedly across regions, with Andean Latin America showing the highest rate (2.01 [95% UI 0.73–3.63] per 100,000 population), while Australasia recorded the lowest (0.24 [95% UI 0.10–0.41] per 100,000 population). The five countries or territories with the highest ASMR were American Samoa (6.66 [95% UI 1.99–12.21] per 100,000 population), Niue (4.89 [95% UI 1.31–10.74] per 100,000 population), Fiji (4.81 [95% UI 1.61–8.95] per 100,000 population), Northern Mariana Islands (4.30 [95% UI 1.38–7.69] per 100,000 population), and Nauru (4.19 [95% UI 1.18–8.77] per 100,000 population), as illustrated in **Figure 2A** and **Table 1**.

#### 3.1.2 SDI variations analyses

**Figure 3:**
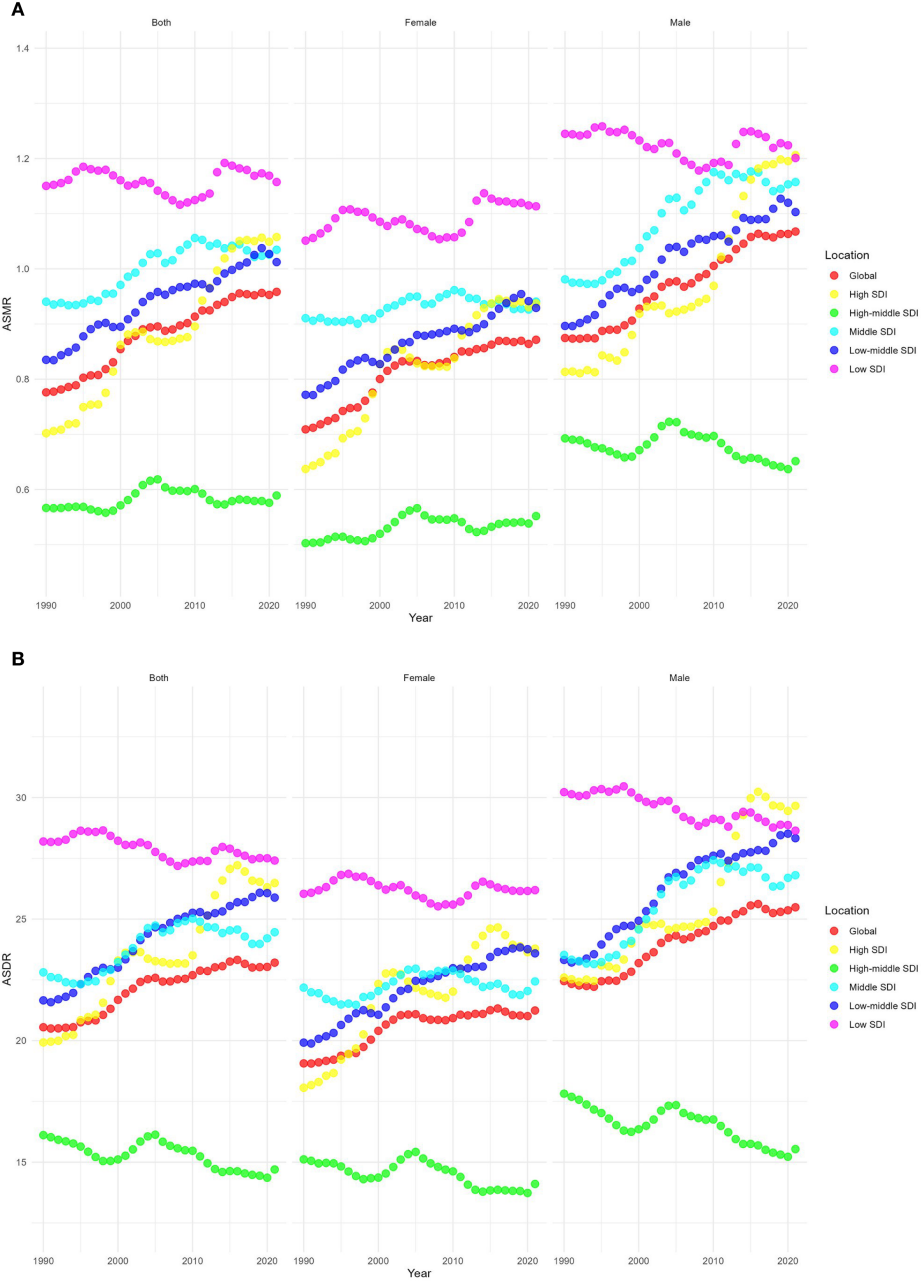
Temporal trends of age-standardized rates of CKD-T2DM-DR across different SDI quintiles. **(A)** ASMR; **(B)** ASDR. CKD-T2DM-DR, chronic kidney disease due to type 2 diabetes mellitus caused by dietary risks; SDI, socio-demographic index; ASMR, age-standardized mortality rate; ASDR, age-standardized disability adjusted life years rate.

From 1990 to 2021, the ASMR of the five SDI regions showed upward trends. Among them, the ASMR grew fastest in the high SDI regions, with an EAPC of 1.4 (95% CI 1.23–1.58), whereas the ASMR grew slowest in the high-middle SDI and low SDI regions, with EAPCs of 0.06 (95% CI -0.17 to 0.29) and 0.01 (95% CI -0.1 to 0.13), respectively. In 2021, the highest ASMR was 1.16 (95% UI 0.44–2.02) per 100,000 population in the low SDI regions, and the lowest was 0.59 (95% UI 0.24–0.97) per 100,000 population in the high-middle SDI regions, as shown in **Figure 3A**.

**Figure 4:**
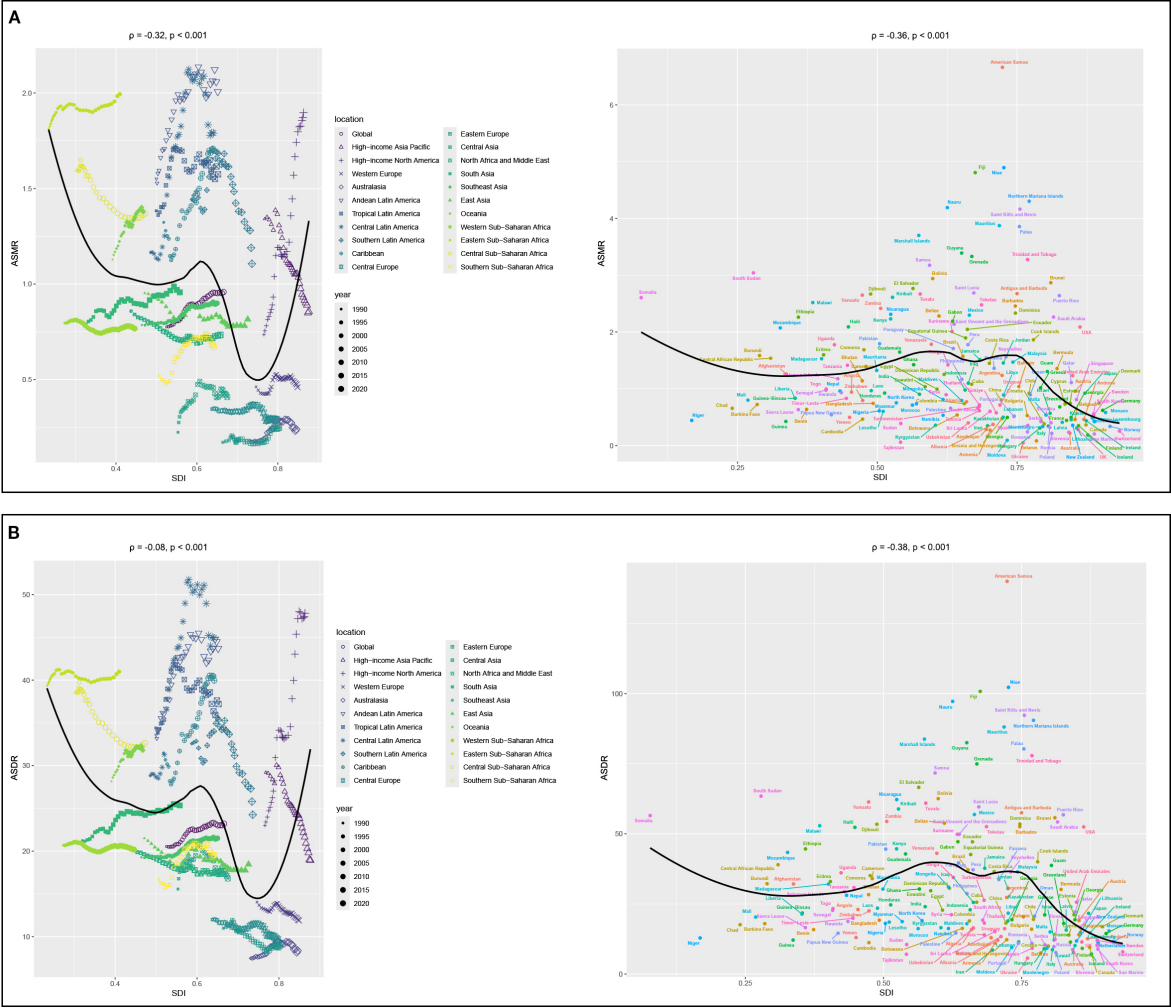
Correlation analyses between age-standardized rates of CKD-T2DM-DR and SDI from 1990 to 2021. **(A)** ASMR; **(B)** ASDR. CKD-T2DM-DR, chronic kidney disease due to type 2 diabetes mellitus caused by dietary risks; SDI, socio-demographic index; ASMR, age-standardized mortality rate; ASDR, age-standardized disability-adjusted life years rate; ρ, Spearman correlation coefficient. A ρ value closer to 1 indicates a strong positive monotonic relationship, whereas a value closer to −1 reflects a strong negative monotonic relationship.

**Figure 5:**
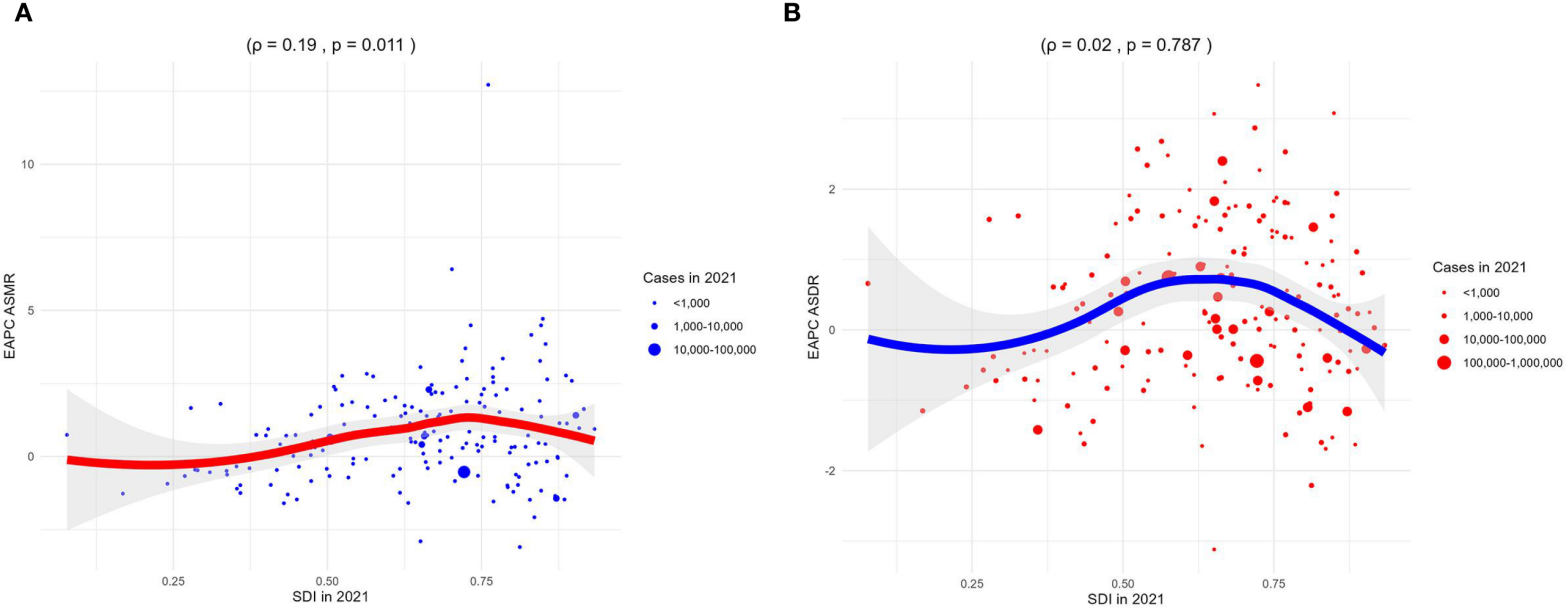
Correlation analyses between EAPCs of CKD-T2DM-DR and SDI in 2021. **(A)** EAPC of ASMR; **(B)** EAPC of ASDR. CKD-T2DM-DR, chronic kidney disease due to type 2 diabetes mellitus caused by dietary risks; SDI, socio-demographic index; EAPCs, estimated annual percentage changes; ASMR, age-standardized mortality rate; ASDR, age-standardized disability-adjusted life years rate; ρ, Spearman correlation coefficient. A ρ value closer to 1 indicates a strong positive monotonic relationship, whereas a value closer to −1 reflects a strong negative monotonic relationship.

Correlation analyses showed that the ASMR of 22 regions (*ρ* = -0.32, *p<* 0.001) and 204 countries or territories (*ρ* = -0.36, *p<* 0.001) was negatively correlated with SDI, as shown in **Figure 4A**. However, a positive correlation was observed between the EAPC of ASMR and the SDI (*ρ* = 0.19, *p* = 0.011), as illustrated in **Figure 5A**.

#### 3.1.3 Gender variations analyses

**Figure 6:**
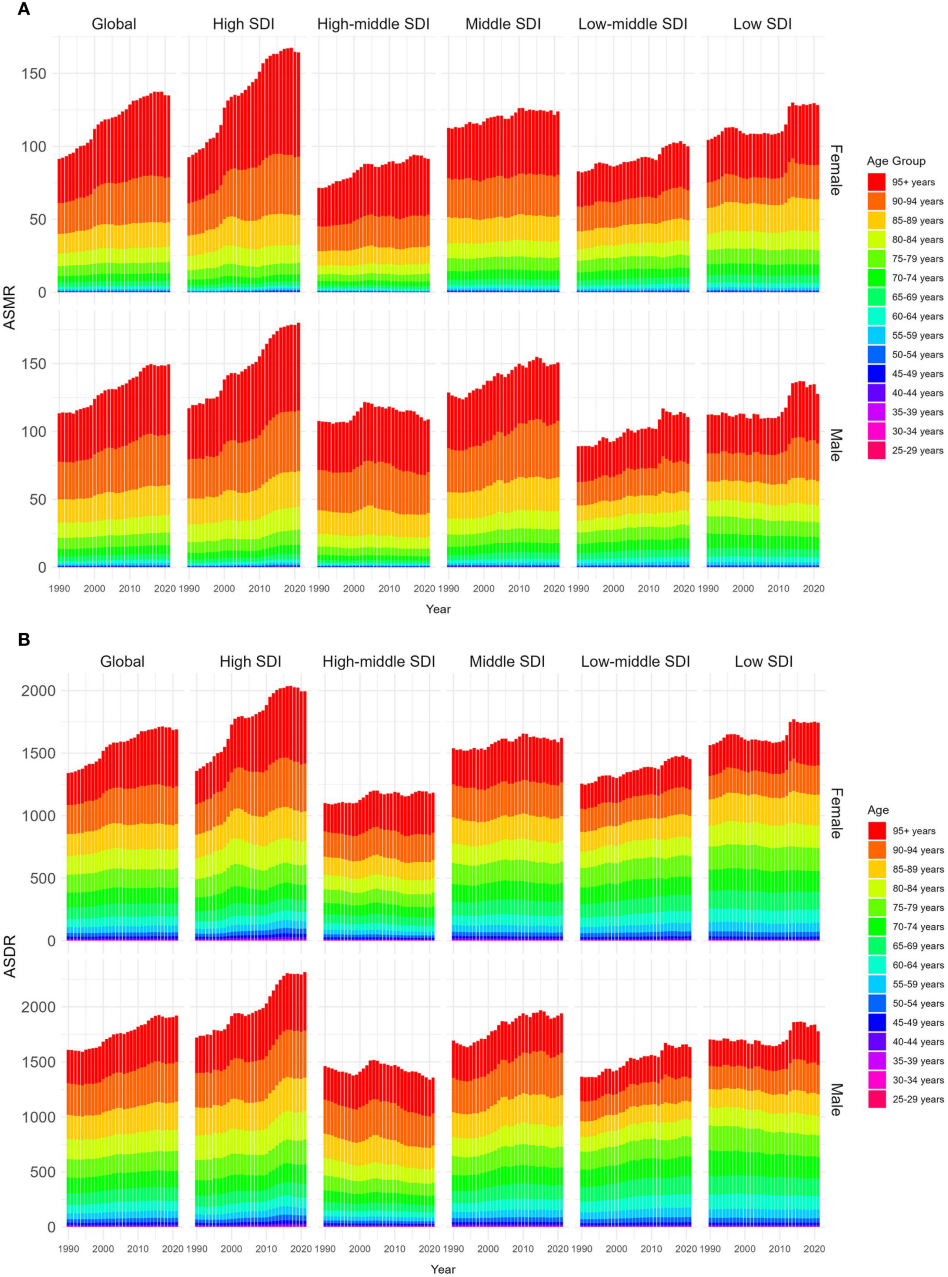
Age-standardized rates of CKD-T2DM-DR by genders and age groups from 1990 to 2021. **(A)** ASMR; **(B)** ASDR. CKD-T2DM-DR, chronic kidney disease due to type 2 diabetes mellitus caused by dietary risks; SDI, socio-demographic index; ASMR, age-standardized mortality rate; ASDR, age-standardized disability-adjusted life years rate.

The ASMR of both males and females showed upward trends, and the ASMR of males was always higher than that of females from 1990 to 2021. In 2021, the global ASMR of males was 1.07 (95% UI 0.44–1.75) per 100,000 population, compared with 0.87 (95% UI 0.36–1.39) per 100,000 in females. The male-to-female ratio of 1.07 vs. 0.87 may be partly attributed to biological differences, as higher testosterone levels in males have been linked to increased susceptibility to renal injury and accelerated disease progression. In the gender-related SDI subgroup analysis, ASMR in high SDI regions grew fastest, both for males and females. Moreover, the ASMR of females in low SDI regions was always higher than that in other SDI regions, while the ASMR of males in high SDI regions was higher than that in other SDI regions since 2021, as depicted **Figure 6A**.

#### 3.1.4 Age variations analyses

From 1990 to 2021, ASMR across all age groups demonstrated upward trends. The ASMR of individuals aged 55–95+ years showed a significant increase with age, with both males and females reaching the highest level at 95+ years. In 2021, the ASMR of males in the 95+ years group was 55.90 (95% UI 18.50–106.00) per 100,000 population, which was higher than the ASMR of females in the same age group (51.44 [95% UI 16.87–99.35] per 100,000 population), as shown in **Figure 6A**.

### 3.2 DALY and ASDR

From 1990 to 2021, the global DALYs of CKD-T2DM-DR increased from 798,255 (95% UI 322,753–1,247,384) to 1,999,209 (95% UI 856,194–3,167,215), and the ASDR increased from 20.55 (95% UI 8.42–32.26) per 100,000 population to 23.21 (95% UI 9.95–36.61) per 100,000 population, with an EAPC of 0.46 (95% CI 0.31–0.60), as presented in **Table 2**.

**Table 2:** DALYs and ASDR of CKD-T2DM-DR and its temporal trends from 1990–2021.

Location	DALYs (95% UI)		ASDR (95% UI)		1990–2021 EAPCs (95% CI)
	1990	2021	1990	2021	
Global	798,255 (322,753–1,247,384)	1,999,209 (856,194–3,167,215)	20.55 (8.42–32.26)	23.21 (9.95–36.61)	0.46 (0.31–0.6)
SDI					
High SDI	219,656 (102,017–333,642)	541,197 (234,392–830,248)	19.92 (9.21–30.27)	26.48 (11.5–40.29)	1.02 (0.86–1.19)
High-middle SDI	155,752 (62,286–245,393)	289,164 (117,290–466,915)	16.11 (6.39–25.55)	14.7 (5.96–23.77)	-0.32 (-0.45 to -0.19)
Middle SDI	229,501 (84,923–377,619)	656,536 (265,076–1,054,870)	22.81 (8.4–37.4)	24.46 (9.87–39.17)	0.34 (0.2–0.48)
Low-middle SDI	129,906 (51,276–208,951)	374,361 (156,643–628,274)	21.66 (8.39–35.07)	25.88 (10.81–43.48)	0.67 (0.51–0.82)
Low SDI	62,647 (24,915–103,251)	136,366 (55,996–233,054)	28.19 (11.42–46.2)	27.41 (11.32–46.78)	-0.12 (-0.23 to -0.01)
Central Europe, eastern Europe, and central Asia					
Central Asia	7,363 (3,705–11,216)	14,217 (7,156–21,361)	15.57 (7.85–23.69)	16.8 (8.37–25.05)	-0.08 (-0.35 to 0.18)
Central Europe	17,853 (9,014–26,577)	21,053 (10,852–33,160)	11.99 (6.1–18)	9.62 (4.95–15.1)	-0.59 (-0.97 to -0.22)
Eastern Europe	30,714 (14,090–45,585)	31,971 (15,908–48,330)	10.99 (5.08–16.43)	9 (4.48–13.6)	-1.16 (-1.31 to -1.01)
High income region					
High-income Asia Pacific	57,080 (29,917–84,083)	96,931 (43,803–145,717)	29.2 (15.28–43.02)	18.93 (8.98–28.32)	-1.42 (-1.77 to -1.07)
High-income North America	79,221 (31,924–122,658)	298,149 (114,207–458,525)	23 (9.35–35.74)	47.82 (17.94–73.13)	2.53 (2.36–2.7)
Western Europe	81,559 (37,209–126,684)	117,704 (52,839–183,078)	14.04 (6.41–21.64)	11.89 (5.35–18.39)	-0.42 (-0.64 to -0.19)
Australasia	1,727 (804–2,644)	4,362 (1,937–6,894)	7.47 (3.44–11.52)	8.37 (3.7–13.03)	0.58 (0.44–0.73)
Southern Latin America	14,939 (5,536–24,526)	21,146 (8,613–33,752)	32.57 (12.21–53.52)	24.26 (9.93–39.12)	-0.6 (-0.92 to -0.28)
Latin America and Caribbean					
Andean Latin America	6,714 (2,275–11,718)	25,762 (9,991–45,106)	33.58 (11.37–58.71)	43.69 (16.77–76.52)	0.95 (0.78–1.13)
Caribbean	8,111 (3,347–13,082)	21,572 (9,686–36,121)	31.48 (12.93–50.63)	39.96 (18.02–66.79)	1.31 (0.98–1.64)
Central Latin America	23,581 (10,205–37,506)	114,055 (51,693–183,127)	28.83 (12.59–46.01)	44.94 (20.14–71.41)	1.96 (1.63–2.3)
Tropical Latin America	31,206 (13,629–49,493)	95,994 (41,541–153,250)	34.39 (14.81–54.83)	37.19 (16.04–59.24)	0.16 (-0.2 to 0.52)
North Africa and Middle East					
North Africa and Middle East	34,315 (13,054–59,040)	84,423 (31,865–141,576)	20.37 (7.78–35.35)	17.65 (6.78–29.9)	-0.49 (-0.62 to -0.35)
South Asia					
South Asia	121,185 (49,981–198,223)	377,972 (158,411–645,464)	21.25 (8.66–34.94)	25.33 (10.52–43.1)	0.62 (0.46–0.79)
Southeast Asia, east Asia, and Oceania					
East Asia	175,246 (50,361–311,379)	396,706 (123,406–701,903)	21.51 (6.33–37.83)	18.46 (5.75–32.68)	-0.44 (-0.56 to -0.32)
Oceania	857 (221–1,572)	2,400 (770–4,377)	28.15 (7.59–51.39)	31.79 (9.99–57.16)	0.34 (0.24–0.43)
Southeast Asia	47,644 (14,384–84,805)	140,942 (44,682–259,240)	19.28 (6.13–34.36)	21.51 (6.85–39.7)	0.46 (0.42–0.5)
Sub-Saharan Africa					
Eastern Sub-Saharan Africa	28,030 (11,332–48,863)	63,948 (27,421–109,061)	39.33 (15.91–69.14)	40.98 (17.42–69.45)	0.01 (-0.11 to 0.14)
Central Sub-Saharan Africa	8,481 (3,188–14,386)	17,806 (6,614–31,898)	38.5 (14–65.95)	32.62 (11.15–58.23)	-0.81 (-1.02 to -0.6)
Southern Sub-Saharan Africa	4,615 (1,800–7,910)	11,656 (4,880–19,762)	16.67 (6.45–29.41)	19.57 (8.21–33.4)	0.78 (0.44–1.11)
Western Sub-Saharan Africa	17,815 (5,876–31,256)	40,439 (15,574–67,102)	20.69 (6.8–35.96)	20.18 (7.41–33.81)	-0.08 (-0.15 to -0.02)

CKD-T2DM-DR, chronic kidney disease due to type 2 diabetes mellitus caused by dietary risks; DALYs, disability-adjusted life years; ASDR, age-standardized DALY rate; EAPCs, estimated annual percentage changes. When EAPC > 0, ASMR or ASDR tends to increase; when EAPC< 0, ASMR or ASDR tends to decrease.

#### 3.2.1 Regional variations analyses

From 1990 to 2021, the ASDR in high-income North America increased the most rapidly, with an EAPC of 2.53 (95% CI 2.36–2.7), whereas the ASDR in high-income Asia Pacific decreased the fastest, with an EAPC of -1.42 (95% CI -1.77 to -1.07), as depicted in **Figure 1B** and **Table 2**. In 2021, ASDR varied markedly across regions, with high-income North America showing the highest rate (47.82 [95% UI 17.94–73.13] per 100,000 population), while Australasia recorded the lowest (8.37 [95% UI 3.70–13.03] per 100,000 population). The top five countries or territories with the highest ASDR were American Samoa (140.02 [95% UI 43.91–257.50] per 100,000 population), Niue (102.23 [95% UI 28.29–222.74] per 100,000 population), Fiji (100.78 [95% UI 33.06–187.59] per 100,000 population), Nauru (97.22 [95% UI 27.87–196.07] per 100,000 population), and Saint Kitts and Nevis (92.25 [95% UI 36.08–165.08] per 100,000 population), as shown in **Figure 2B** and **Table 2**.

#### 3.2.2 SDI variations analyses

From 1990 to 2021, the ASDRs of high SDI, middle SDI, and low-middle SDI regions showed upward trends, whereas the ASDRs of high-middle SDI and low SDI regions showed downward trends. The regions with the most rapid increase in ASDR were those with high SDI, with an EAPC of 1.02 (95% CI 0.86–1.19), whereas the regions with the fastest decline were those with high-middle SDI, with an EAPC of -0.32 (95% CI -0.45 to -0.19). In 2021, the regions with the highest ASDR were those with low SDI (27.41 [95% UI 11.32–46.78] per 100,000 population), whereas the region with the lowest ASDR were those with high-middle SDI (14.7 [95% UI 5.96–23.77] per 100,000 population), as depicted in **Figure 3B**.

Correlation analyses revealed that the ASDR of 22 regions (*ρ* = -0.08, *p* < 0.001) and 204 countries or territories (*ρ* = -0.38, *p* < 0.001) was negatively with SDI, as shown in **Figure 4B**. Furthermore, the EAPC of ASDR was not related to the SDI in 2021 (*ρ* = 0.02, *p* = 0.787), as shown in **Figure 5B**.

#### 3.2.3 Gender variations analyses

From 1990 to 2021, the ASDR for both males and females showed upward trends, and the ASDR of males was higher than that of females. In 2021, the global ASDR of males was 25.49 (95% UI 10.72–40.90) per 100,000 population, which was higher than that of females (21.24 [95% UI 9.13–33.47] per 100,000 population). The male-to-female ratio of 25.49 vs. 21.24 may be partly attributed to biological differences, as higher testosterone levels in males have been linked to increased susceptibility to renal injury and accelerated disease progression. In the gender-related SDI subgroup analyses, the ASDR in high SDI regions increased most rapidly, regardless of males or females. Additionally, the ASDR of females in low SDI regions has always been higher than that in other SDI regions, while the ASDR of males in high SDI regions has always been higher than that in other SDI regions since 2015, as shown in **Figure 6B**.

#### 3.2.4 Age variations analyses

From 1990 to 2021, ASDRs showed upward trends in all age groups. The ASDR for those aged 55 to 95 years has a significant trend with age, and the ASDR of both male and female reaches the highest at 95+ years. In 2021, the ASDR for males aged 95+ years is 456.17 (95% UI 152.40–861.86) per 100,000 population, which is higher than that for females (422.20 [95% UI 138.71–811.79] per 100,000 population), as shown in **Figure 6B**.

### 3.3 Analyses of dietary risk factors

**Figure 7:**
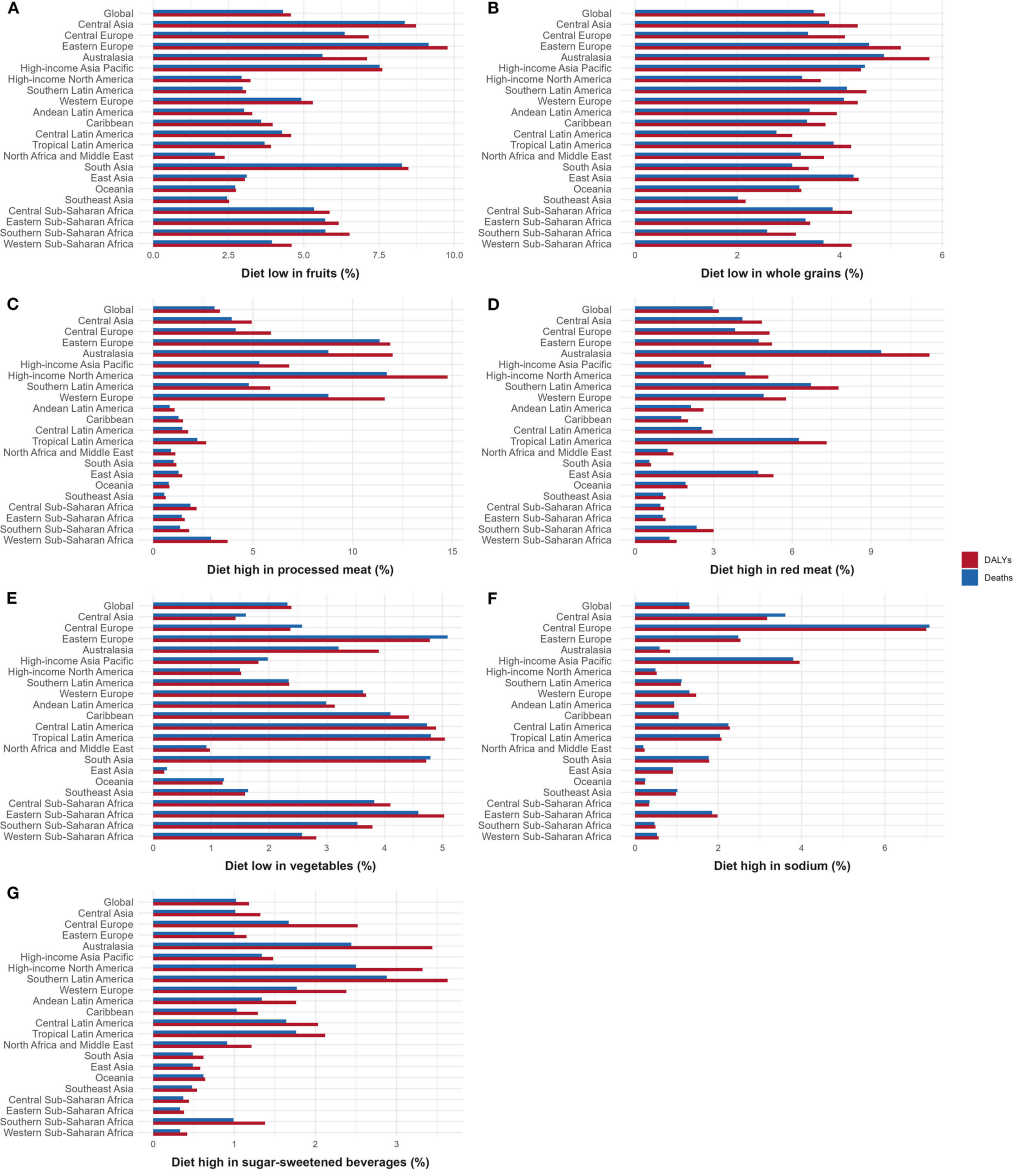
Contribution of different dietary risks to CKD-T2DM-DR in 2021. **(A)** Diet low in fruits; **(B)** Diet low in whole grains; **(C)** Diet high in processed meat; **(D)** Diet high in red meat; **(E)** Diet low in vegetables; **(F)** Diet high in sodium; **(G)** Diet high in sugar-sweetened beverages. CKD-T2DM-DR, chronic kidney disease due to type 2 diabetes mellitus caused by dietary risks; DALYs, disability-adjusted life years.

From 1990 to 2021, seven dietary risks contributed differently to the global burden of CKD-T2DM. Ranking these risks by their impact on death and DALY percents, diet low in fruit was the leading contributor (death: 4.31%; DALY: 4.57%), followed by diet low in whole grains (death: 3.49%; DALY: 3.71%), diet high in processed meat (death: 3.08%; DALY: 3.35%), diet high in red meat (death: 2.96%; DALY: 3.20%), diet low in vegetables (death: 2.32%; DALY: 2.39%), diet high in sodium (death: 1.30%; DALY: 1.31%), and diet high in sugar-sweetened beverages (death: 1.02%; DALY: 1.18%). This ranking provides a clear hierarchy of dietary risks and highlights the relative contributions of each factor to the global burden of CKD-T2DM, as further illustrated in **Figure 7**.

#### 3.3.1 Diet low in fruit

Globally, the DALY and death percents of CKD-T2DM caused by diet low in fruit were 4.57% and 4.31%, respectively. In Eastern Europe, this dietary risk was most prominent, contributing 9.15% of deaths and 9.78% of DALYs. North Africa and the Middle East had the lowest percentages, with 2.05% for deaths and 2.37% for DALYs, as illustrated in **Figure 7A**.

#### 3.3.2 Diet low in whole grains

Globally, the DALY and death percents of CKD-T2DM caused by diet low in whole grains were 3.71% and 3.49%, respectively. Regionally, Australasia had the highest percentages, with 4.86% for deaths and 5.75% for DALYs, and Southeast Asia had the lowest percentages, with 2.01% for deaths and 2.16% for DALYs, as depicted in **Figure 7B**.

#### 3.3.3 Diet high in processed meat

Globally, the DALY and death percents of CKD-T2DM caused by diet high in processed meat were 3.35% and 3.08%, respectively. Among them, high-income North America exhibited the greatest impact, with 11.71% of deaths and 14.77% of DALYs. South Asia had the lowest contributions, with 1.02% for deaths and 1.16% for DALYs, as shown in **Figure 7C**.

#### 3.3.4 Diet high in red meat

Globally, the DALY and death percents of CKD-T2DM caused by diet high in red meat were 3.20% and 2.96%, respectively. Regionally, Australasia showed the highest impact, with 9.39% of deaths and 11.23% of DALYs. South Asia recorded the lowest percentages, with 0.55% for deaths and 0.61% for DALYs, as illustrated in **Figure 7D**.

#### 3.3.5 Diet low in vegetables

Globally, the DALY and death percents of CKD-T2DM caused by diet low in vegetables were 2.39% and 2.32%, respectively. Eastern Europe had the highest deaths percent at 5.09%, whereas Tropical Latin America had the highest DALYs percent at 5.04%. East Asia recorded the lowest percentages, with 0.24% for deaths and 0.19% for DALYs, as shown in **Figure 7E**.

#### 3.3.6 Diet high in sodium

Globally, the DALY and death percents of CKD-T2DM caused by diet high in sodium were 1.31% and 1.30% respectively. Regionally, Central Europe exhibited the highest percentages, with 7.07% of deaths and 6.99% of DALYs. North Africa and the Middle East reported the lowest figures, with 0.20% for deaths and 0.23% for DALYs, as depicted in **Figure 7F**.

#### 3.3.7 Diet high in sugar-sweetened beverages

Globally, the DALY and death percents of CKD-T2DM caused by diet high in sugar-sweetened beverages were 1.18% and 1.02% respectively. Southern Latin America recorded the highest percentages, with 2.88% for deaths and 3.63% for DALYs. Eastern Sub-Saharan Africa had the lowest figures, with 0.33% for deaths and 0.38% for DALYs, as shown in **Figure 7G**.

## 4 Discussion

### 4.1 Research significance and key findings

This study offers a thorough assessment of DALY and mortality of CKD-T2DM-DR and investigates its trends over time and influencing factors globally. The key findings are as follows: First, from 1990 to 2021, the mortality, DALYs, ASDR, and ASMR of CKD-T2DM-DR were increasing globally. Second, the ASDR and ASMR of CKD-T2DM-DR were negatively correlated with SDI. Third, the growth rate of ASMR for CKD-T2DM-DR was positively correlated with SDI, whereas the growth rate of ASDR for CKD-T2DM-DR was not correlated with SDI. Fourth, the ASDR and ASMR of CKD-T2DM-DR increased with age, and were higher in males than in females. Fifth, the DALY and death percents of CKD-T2DM due to diet low in fruit were the highest, whereas the DALY and death percents of CKD-T2DM due to diet high in sugar-sweetened beverages were the lowest.

### 4.2 Global and regional burden analysis

The burden of CKD-T2DM-DR demonstrated an increasing trend from 1990 to 2021. Globally, the ASMR for CKD-T2DM-DR increased from 0.78 per 100,000 population to 0.96 per 100,000 population, whereas the ASDR increased from 20.55 per 100,000 population to 23.21 per 100,000 population. This alerts us that the health burden of CKD-T2DM-DR is progressively intensifying, necessitating increased attention to the epidemiological trend of CKD-T2DM-DR and develop rational prevention and treatment.

In 2021, high-income North America recorded the highest ASDR, whereas Andean Latin America exhibited the highest ASMR, suggesting that the burden of CKD-T2DM-DR is relatively severe in North and South America. In contrast, Australasia is the region with the lowest ASDR and ASMR, showing a clear geographic difference. Furthermore, the growth rates of ASDR and ASMR in high-income North America were the most rapid, implying that high-income North America may be a major current and future disaster area for CKD-T2DM-DR. Therefore, health care departments in high-income North America need to focus on the diagnosis and treatment of CKD-T2DM-DR and adopt more aggressive prevention and control policies. Interestingly, ASDR and ASMR declined fastest in the high-income Asia Pacific region, possibly benefiting from its inherent dietary patterns and the economic development over the past thirty years. In fact, the differences between regions may be the combined effects of multiple factors such as economic level, healthcare system, demographic structures, and dietary habits. These findings carry profound implications for policy, especially in the global quest to address the burden of CKD-T2DM-DR.

### 4.3 Burden analysis based on SDI

Correlation analyses revealed that ASDR and ASMR for CKD-T2DM-DR were negatively correlated with SDI, with low SDI regions exhibiting the highest burden in 2021. This pattern reflects the dual challenges faced by these regions: poor dietary access and inadequate healthcare. For one, low economic development and household income restrict the ability to afford nutritious diets, increasing CKD-T2DM-DR risk. For another, weak healthcare systems and limited medication availability hinder early detection and optimal management, resulting in many patients not receiving timely and effective treatment. For example, the prevalence of DKD in sub-Saharan Africa ranges from 6% to 16%, but access to dialysis treatment is less than 20 per million population, and in some areas dialysis is not performed at all (15). In contrast, high-middle SDI regions recorded the lowest ASDR and ASMR, likely due to a stronger economic base compared with low SDI regions and relatively lower aging levels compared with high SDI regions. These findings indicate that economic foundation, healthcare system capacity, and population aging contribute to SDI-related disparities in CKD-T2DM-DR burden. It is also important to note that genetic susceptibility and environmental exposures may further influence CKD-T2DM-DR risk in low SDI regions. However, the GBD estimates used in this study account for these confounding factors, and they are not the primary focus of our analysis.

Correlation analyses showed that the growth rate of ASMR for CKD-T2DM-DR was positively correlated with SDI, while the growth rate of ASDR was not associated with SDI. Specifically, high SDI regions experienced the most rapid increases in both ASDR and ASMR from 1990 to 2021, primarily driven by population aging and westernized diets. Advances in healthcare have markedly prolonged life expectancy in these regions, thereby increasing the proportion of older adults. For instance, a study in Sweden reported that between 2009 and 2015, the population increased by 510,335 individuals, with those aged 65 to 84 years accounting for 17.1% of the increase (16). Given that aging is a critical risk factor for DKD (17), population aging inevitably enlarges the high-risk demographic for CKD-T2DM-DR, contributing to a rise in its burden. In addition, the growing prevalence of westernized dietary patterns, which are characterized by high caloric intake, consumption of processed foods, and excessive sodium, accelerates obesity, hypertension, and the progression of diabetes, thereby further amplifying the public health challenge posed by CKD-T2DM-DR. Furthermore, ASDR is also increasing in middle SDI and low-middle SDI regions, likely due to fragile economic foundations and imperfect healthcare systems. For example, Perkovic et al. (18) reported that the prevalence of stage III CKD was significantly higher in economically disadvantaged rural regions than in urban regions in Thailand. Although Thailand has implemented universal health coverage since 2002, healthcare services still vary across socioeconomic groups due to disparities in economic development and distribution (19).

The growth of ASMR in high-middle SDI regions was the slowest from 1990 to 2021, with a decreasing trend in ASDR. This may be owing to the ongoing economic development and constantly improving healthcare systems in these regions, as well as a less pronounced aging population compared to high SDI regions. Furthermore, low SDI regions also presented a slow growth in ASMR and a negative growth in ASDR, which is also attributed to the gradual improvement of the economy and the health care system. For example, since Nepal implemented a nationwide free healthcare policy in 2008, a greater number of Nepali citizens have gained access to basic medical services (20). With economic development and improvement of transportation convenience, more Nepalis are actively seeking treatment at public healthcare institutions (21). However, despite the continuous enhancement of health care systems and public health policies in low SDI regions, these regions still exhibit the highest ASDR and ASMR for CKD-T2DM-DR. This indicates that low SDI regions continue to be the main disaster region of CKD-T2DM-DR, and their medical level and health care system need to be further improved. In conclusion, the burden of CKD-T2DM-DR is intricately linked to SDI, with this relationship largely shaped by economic level, health care system, and population aging. Therefore, individualized prevention and control strategies for CKD-T2DM-DR need to be formulated for different SDI regions.

### 4.4 Burden analyses based on gender and age

In gender and age-based analyses, the present study demonstrated that ASMR and ASDR for CKD-T2DM-DR were higher in the male and elderly population. As significant risk factors for DKD, male gender, smoking and alcohol consumption are strongly associated with the prognosis of CKD-T2DM-DR, collectively contributing to a larger burden of CKD-T2DM-DR in males (22, 23). For one thing, androgen secretion in males aggravates renal impairment. A cross-sectional Korean study showed that total testosterone levels were significantly higher in patients with DKD than in diabetic patients without nephropathy, and identified a positive relationship between urinary protein and total testosterone levels (24). A subsequent animal study confirmed that testosterone aggravated renal impairment by inducing podocyte apoptosis (25). These findings emphasize that higher levels of testosterone in males are detrimental to the prognosis of DKD. On the contrary, estrogen is known for antioxidant, anti-inflammatory, and vascular endothelial protective properties, which are thought to play a positive role in protecting the kidney (26, 27). A clinical study in Hungary noted that oral oestradiol and norethindrone significantly improved proteinuria and creatinine clearance in postmenopausal females with diabetes and hypertension (28). This suggests that differences in sex hormone levels between males and females may influence the burden of CKD-T2DM-DR. For the other thing, smoking and alcohol consumption, the main risk factors for DKD, are more prevalent among males. It is reported that in 2020, about 22.3% of the global population smoked, and the prevalence of smoking was notably higher in males than in females (36.7% *vs*. 7.8%) (29). Additionally, in 2019, the prevalence of alcohol consumption (52% *vs*. 35%) and the volume of alcohol consumed (8.2 litres *vs*. 2.2 litres) were significantly higher in males than in females worldwide (30). These findings reveal the role of smoking and alcohol consumption in increasing the burden of CKD-T2DM-DR in males.

Moreover, aging constitutes an equally significant risk factor for DKD (17), which explains why the elderly population possesses a larger burden of CKD-T2DM-DR. A previous study showed that aging is an independent predictive risk factor for DKD (OR 1.14, 95% CI 1.09–1.19) (31). Subsequent research highlighted that the inflammation and oxidative stress, which are progressively exacerbated by aging, are pivotal contributors to the deterioration of renal function (32). In conclusion, the development of rational dietary interventions and control strategies is extremely crucial to alleviate the burden in patients with CKD-T2DM-DR, especially in males and elderly patients.

### 4.5 Burden analyses based on different dietary risks

In the dietary risk analysis, the DALY and death percents of CKD-T2DM caused by diet low in fruit were the highest. This may be due to the fact that fruits contain high levels of trace elements and other beneficial components, the deficiency of which increases the risk of diabetes and its complications. Previous studies showed that flavonoids present in a variety of fruits are effective in improving renal function (33–35). For example, fisetin attenuated high glucose-induced podocyte injury by modulating the autophagy-dependent cyclin-dependent kinase inhibitor 1B/p70 ribosomal protein S6 kinase (CDKN1B/p70S6K) signaling pathway and suppressing the NOD-like receptor family pyrin domain containing 3 (NLRP3) inflammasome activation (33). In addition, fisetin activated the renal nuclear factor erythroid 2-related factor 2/heme oxygenase-1/glutathione peroxidase 4 (Nrf2/HO-1/GPX4) pathway, thereby enhancing antioxidant defenses and alleviating podocyte injury in DKD mice (34). Moreover, naringin was reported to exert renoprotective effects by attenuating oxidative stress and mitigating mitochondrial dysfunction (35). These findings imply the significance of adequate fruit intake in reducing the burden of CKD-T2DM. Furthermore, the DALY and death percents of CKD-T2DM caused by diet high in sugar-sweetened beverages were the lowest. This may be attributed to dietary management of diabetes and initiatives by the World Health Organization (WHO) to reduce the amount of added sugar. For one thing, patients with diabetes actively limit their sugar intake to manage their condition. For another, the WHO, in its Guideline: Sugars Intake for Adults and Children, emphasizes that the intake of added sugars should be limited to less than 10% of the total energy intake, and preferably less than 5% (36). These efforts have brought the burden of CKD-T2DM caused by diet high in sugar-sweetened beverages under some control.

Given the marked geographic differences in the burden of CKD-T2DM-DR, we further analyzed the reasons for the differences in the distribution of each CKD-T2DM-DR. Whole grains are rich in dietary fibre and represent a crucial source of nutrition. Previous studies have found a negative association between fibre intake and mortality in patients with kidney disease (37), suggesting an association between whole grain intake and the burden of CKD-T2DM. Our study showed that the region with the highest DALY and death percents for CKD-T2DM caused by diet low in whole grains was Australasia, and the region with the lowest was Southeast Asia. This difference may be due to the dietary structure itself. An Australian health survey indicated that 73% of individuals aged 9 and above fail to meet the recommended daily intake of 48 g of whole grains (38). Of these, the median daily intake of whole grains for the 19 to 85 years old group was only 21g (38). In contrast, Southeast Asia, a region abundant in grain production, benefits from a rich supply of whole grains, which significantly reduces the burden of CKD-T2DM caused by diet low in whole grains. Therefore, we expect that regions represented by Australia advise patients to increase whole grain intake in the prevention and management strategies for CKD-T2DM.

Vegetables are rich in a variety of trace elements and other beneficial components, which have positive effects on human health. Previous studies indicated that vegetable diets reduce the risk of impaired kidney function and CKD (39), suggesting a link between vegetable intake and the burden of CKD-T2DM. Our study showed that the region with the highest death of CKD-T2DM caused by diet low in vegetables was Eastern Europe and the region with the highest DALYs was Tropical Latin America, whereas these with the DALY and death were lowest in East Asia. This difference may be related to dietary patterns. WHO recommends a combined daily intake of more than 400 g of fruits and vegetables, with vegetables alone constituting more than 240g (40). However, in Latin America, there is a high intake of animal products and sugar, but a markedly insufficient and steadily declining intake of vegetables and fruits (41). Additionally, Eastern European countries, represented by Russia, also have a significant deficit in fruit and vegetable intake (40). It has been reported that only 49% of males and 60% of females in Russia meet the recommended daily intake of fruits and vegetables (40). In contrast, daily vegetable intake in Eastern Asia is as high as 349 g, far exceeding the WHO recommended minimum of 240 g (42). Therefore, we propose that in the preventive and treatment strategies for CKD-T2DM, regions represented by Eastern Europe and tropical Latin America advise patients to increase their vegetable intake.

Fruits are rich in vitamins and trace elements with anti-inflammatory and antioxidant properties (43). Previous studies demonstrated that the risk of CKD decreased with increased fruit intake (44), suggesting a link between fruit intake and the burden of CKD-T2DM. Our study showed that the highest DALY and death of CKD-T2DM caused by diet low in fruit were in Eastern Europe, and the lowest in Middle East and North Africa. This difference may be due to the dietary structure itself. Russia, the most populous country in Eastern Europe, has a diet structure that is dominated by high carbohydrates, proteins, and fats, with a markedly low intake of vegetables and fruits (40). In contrast, the structure of the diet in Middle East and North Africa is dominated by grains, fruits, and vegetables (45). Although the total structure of food consumption in the Middle East and North Africa regions is shifting towards modern food consumption patterns, the Mediterranean diet rich in fruits and vegetables still holds an important place (45). Therefore, we suggest that patients in regions represented by Eastern Europe be advised to increase their fruit intake in the prevention and treatment strategies for CKD-T2DM.

Sugar-sweetened beverages are typically rich in simple carbohydrates and added sugars, which can lead to poor glycaemic control, insulin resistance, and chronic inflammation (46). Previous studies revealed that sugar-sweetened beverages were correlated with an increased risk of DKD (47), suggesting a link between high sugar-sweetened beverage intake and the burden of CKD-T2DM. Our study showed that the region with the highest DALY and death of CKD-T2DM caused by diet high in sugar-sweetened beverages was Southern Latin America, and that with the lowest was Eastern Sub-Saharan Africa. This may be the result of a combination of dietary structure and economic level. For one thing, Southern Latin America is the region with the highest consumption of sugar-sweetened beverages (48). Globally, three of the six countries with the highest reported sales of sugary beverages per capita per day in kcal or bottle cap are located in Southern Latin America, including Chile, Argentina, and Peru (48). In contrast, the Eastern Sub-Saharan Africa region has a high reliance on staple foods such as vegetables, grains, and pulses, while consumption of sugary beverages remains relatively low (48). For another, in low- and middle-income countries, increased consumption of sugar-sweetened beverages is correlated with urbanization and economic growth (49). As Latin America contains many low- or middle-income countries, their consumption of sugar-sweetened beverages may be correlated with economic growth and transition. Conversely, residents of Eastern Sub-Saharan Africa, where economic levels are limited, tend to favour the consumption of food essentials rather than sugary drinks. Relevant studies confirmed that in Kenya and Burkina Faso, the consumption of sugar-sweetened beverages among females of childbearing age was positively associated with household income and negatively associated with household food insecurity (50). Therefore, we suggest that in preventive and treatment strategies for CKD-T2DM, regions such as Southern Latin America advise patients to reduce the consumption of sugary beverages.

Sodium is a vital trace element for the human body, as it plays an essential role in maintaining normal cellular function, acid-base balance, plasma volume, and nerve conduction. However, diets high in sodium induce renal fibrosis and endothelial dysfunction through oxidative stress and inflammatory responses, which in turn lead to deterioration of renal function (51–53). Our study indicated that the region with the highest DALY and death of diet high in sodium-related CKD-T2DM was Central Europe, and whose with the lowest were North Africa and Middle East. This difference also originates from dietary structure. Sodium intake is generally high in Central Europe, particularly in Poland, where 73.4% of the population is reported to consume excessive amounts of salt (54). In contrast, the North Africa and Middle East regions are dominated by a Mediterranean diet rich in grains, legumes, and fruits and vegetables, and is well flavored with herbs and spices, resulting in a relatively low consumption of salt (55, 56). Therefore, we recommend that a low-sodium diet be included in the preventive and treatment strategies for CKD-T2DM in the region represented by Central Europe.

Red meat serves as a significant source of high-quality protein, zinc, and B vitamins, but it is also rich in saturated fat and other unhealthy components. Relevant studies indicated that nitrite, nitrate, heme iron, and advanced glycation end products in red meat contribute to oxidative stress and insulin resistance (57, 58). Subsequent studies revealed a positive association between red meat intake and the risk of DKD (59), suggesting a link between a diet high in red meat and the burden of CKD-T2DM. Our study showed that the region with the highest DALY and death for diet high in red meat-related CKD-T2DM was Australasia, and that with the lowest was South Asia. This difference is also thought to arise from regional dietary structures. Australia and New Zealand, major global producers and exporters of red meat, place it at the heart of their culinary traditions. Additionally, owing to cultural factors, red meat is often a key part of family gatherings and traditional festivals in these regions. In contrast, the diet in South Asia is dominated by plant-based foods such as vegetables, pulses, and grains, with a reduced intake of red meat. The average global consumption of unprocessed red meat in 2018 was reported to be 51 g/day, whereas South Asia ranked at the bottom of the list with an average consumption of 7 g/day (60). Therefore, we recommend that regions represented by Australasia include limiting red meat intake in the prevention and treatment strategy for CKD-T2DM.

Processed meats contain high levels of sodium chloride, nitrites, and other harmful substances. Previous studies demonstrated that high consumption of processed meat is linked to poor prognosis of DKD (61), suggesting a connection between processed meat diet and the burden of CKD-T2DM. Our study revealed that the region with the highest DALY and death for diet high in processed meat-associated CKD-T2DM was high-income North America, and the region with the lowest was South Asia. This difference depends on dietary structure and economic level. For one thing, the high-income North American region has the highest intake of processed meat in the world, far exceeding the global average (62). For another thing, high-income North America has a solid economic and industrial base to build and maintain a complete processed meat industry chain; moreover, policies such as the North American Free Trade Agreement have encouraged the production, trade, and consumption of agricultural products, including processed meat (63). Conversely, South Asia has the lowest average consumption of processed meat in the world, at 3 g/day, owing to its dietary structure and economic level (60). Therefore, we recommend that regions represented by high-income North America include limiting processed meat intake in the prevention and treatment strategies for CKD-T2DM.

### 4.6 Limitations and prospects

Our study has several limitations. First, although the GBD database includes data from 204 countries and territories worldwide, the completeness and accuracy of these data may vary by region, particularly in low- and middle-income countries where underreporting or incomplete data may introduce bias. Second, discrepancies between GBD disease classifications and other international standards may affect the precision of disease burden estimates. Third, advances in diagnostic techniques and healthcare systems over time may compromise the comparability of data, potentially biasing long-term trend analyses. Fourth, although the GBD database adjusted for conventional demographic and environmental factors, the data did not allow for full control of potential confounding factors such as diabetes duration or antihypertensive therapy. Consequently, the observed associations between dietary factors and CKD-T2DM-DR may be influenced by residual confounding, and the results should be interpreted with caution. To address these limitations, future research should include more rigorous assessments of the global and regional burden of CKD-T2DM-DR, incorporating improved disease classification and accounting for diagnostic variability. Additionally, randomized trials testing fruit intake interventions in high-risk populations, as well as longitudinal studies examining dietary shifts and DKD progression, are warranted to provide more definitive evidence for prevention and management strategies.

## 5 Conclusion

From 1990 to 2021, the global burden of CKD-T2DM-DR has been steadily increasing with significant regional variability. Low SDI regions are most severely affected by this challenge, while high SDI regions are experiencing a rapid increase in the burden. The global burden of CKD-T2DM-DR increases with age, and is higher in males than in females. Among the seven dietary risks, the diet low in fruit was identified as the primary dietary risk for CKD-T2DM. These findings emphasize the significance of formulating preventive strategies tailored to diverse regions and populations: low SDI regions would benefit from fruit supplementation programs and initiatives to improve dietary access, while high SDI regions should implement campaigns aimed at reducing intake of processed meats and other high-risk dietary components. In addition, future research should include randomized trials testing fruit intake interventions in high-risk populations and longitudinal studies examining the impact of dietary shifts on CKD progression, to provide more definitive evidence for prevention and management strategies.

## Data Availability

The original contributions presented in the study are included in the article/supplementary material. Further inquiries can be directed to the corresponding author.
